# Aberrant brain network topology in fronto‐limbic circuitry differentiates euthymic bipolar disorder from recurrent major depressive disorder

**DOI:** 10.1002/brb3.1257

**Published:** 2019-05-07

**Authors:** Jannis Dvorak, Marietheres Hilke, Marco Trettin, Sofia Wenzler, Marleen Hagen, Naddy Ghirmai, Maximilian Müller, Dominik Kraft, Andreas Reif, Viola Oertel

**Affiliations:** ^1^ Department of Psychiatry, Psychosomatic Medicine and Psychotherapy Goethe University Frankfurt Frankfurt Germany; ^2^ Brain Imaging Center (BIC) Goethe University Frankfurt Frankfurt Germany

**Keywords:** bipolar disorder, euthymic, fMRI, functional connectivity, graph theory, major depressive disorder, resting‐state

## Abstract

**Introduction:**

Previous studies have established graph theoretical analysis of functional network connectivity (FNC) as a potential tool to detect neurobiological underpinnings of psychiatric disorders. Despite the promising outcomes in studies that examined FNC aberrancies in bipolar disorder (BD) and major depressive disorder (MDD), there is still a lack of research comparing both mood disorders, especially in a nondepressed state. In this study, we used graph theoretical network analysis to compare brain network properties of euthymic BD, euthymic MDD and healthy controls (HC) to evaluate whether these groups showed distinct features in FNC.

**Methods:**

We collected resting‐state functional magnetic resonance imaging (fMRI) data from 20 BD patients, 15 patients with recurrent MDD as well as 30 age‐ and gender‐matched HC. Graph theoretical analyses were then applied to investigate functional brain networks on a global and regional network level.

**Results:**

Global network analysis revealed a significantly higher mean global clustering coefficient in BD compared to HC. We further detected frontal, temporal and subcortical nodes in emotion regulation areas such as the limbic system and associated regions exhibiting significant differences in network integration and segregation in BD compared to MDD patients and HC. Participants with MDD and HC only differed in frontal and insular network centrality.

**Conclusion:**

In conclusion, our findings indicate that a significantly altered brain network topology in the limbic system might be a trait marker specific to BD. Brain network analysis in these regions may therefore be used to differentiate euthymic BD not only from HC but also from patients with MDD.

## 1 INTRODUCTION

Bipolar disorder (BD) and major depressive disorder (MDD) are severe mood disorders often characterized by a perseverative course across the affected individuals' lifetimes (Fountoulakis, 2010; Grande, Berk, Birmaher, & Vieta, 2016; Hardeveld, Spijker, De Graaf, Nolen, & Beekman, 2010; Kessler et al., 2003). Often enough, it is most difficult to distinguish patients with BD and MDD as both disorders manifest themselves highly similar in depressive episodes. This can lead not only to wrong diagnoses but also to inappropriate treatment (Correa et al., 2010). Despite the comparable clinical appearance, efforts have been made to identify BD and MDD patients by their neuropathological differences (Strakowski et al., 2012; Strakowski, Adler, & DelBello, 2002). The search for and evaluation of these biomarkers therefore have become an increasingly emphasized field of research over the past years.

Recent studies have utilized functional magnetic resonance imaging (fMRI) as a potential tool to differentiate BD and MDD (Anand, Li, Wang, Lowe, & Dzemidzic, 2009; Goya‐Maldonado et al., 2016; Liu et al., 2013, 2015; Marchand, Lee, Johnson, Gale, & Thatcher, 2013; Rive et al., 2016; Sacchet, Livermore, Iglesias, Glover, & Gotlib, 2015; Wang et al., 2015). Especially resting‐state fMRI (r‐fMRI) has gained increased attention because of its ability to monitor spontaneous hemodynamic responses that are task‐independent without application of external stimuli (Lee, Smyser, & Shimony, 2013). Various studies were able to find aberrant resting‐state brain activation patterns in BD and MDD compared with healthy controls (HC) using r‐fMRI (Mulders, van Eijndhoven, Schene, Beckmann, & Tendolkar, 2015; Vargas, López‐Jaramillo, & Vieta, 2013). Usually, activation schemes of two or more brain regions are correlated with each other to obtain information about their functional connectivity (FC) (Friston, 2002). This enables the detection of functional brain networks underlying various cognitive functions and dysfunctions.

Anand et al. (2009) conducted the first study to compare bipolar and unipolar depressed patients using FC analysis on resting‐state fMRI data. They found decreased FC in the corticolimbic network in both mood disorders compared to healthy individuals with more severe decreases in the BD group. Other studies (e.g. Goya‐Maldonado et al., 2016; Wang, Wang, Jia, Zhong, Zhong et al., 2017) observed differences between patients and HC as well as between BD and MDD using a diversity of FC analysis procedures. Most studies showed differences between affective disorders and HC mainly in the limbic circuitry as well as in prefrontal regions which are known to have an impact on the emotion regulation process (Blond, Fredericks, & Blumberg, 2012; Strakowski et al., 2012).

Recently, graph analysis using graph theoretical measures has been applied to explore brain network properties in individuals with psychiatric disorders (Bassett & Bullmore, 2009; He et al., 2016; Manelis et al., 2016; Wang, Wang, Jia, Zhong, Niu et al., 2017; Wang, Wang, Jia, Zhong, Zhong et al., 2017). Using graph analysis, the brain is modulated as a network of nodes (most commonly a priori defined regions) that are connected by edges resembling functional connections between these regions (Bassett & Bullmore, 2006, 2017). Graph theory (GT) can be applied to investigate both global network changes and alterations only affecting distinct regions and is ideally suited for studying complex networks such as the human brain (Fornito, Zalesky, & Breakspear, 2013). As an outstandingly complex network, the brain features maximum efficiency while minimizing costs of information processing (Bassett & Bullmore, 2006). GT tools assist in examining functional interactions between brain regions and evaluating their underlying network architecture without having to narrow the view to a predefined set of regional connections (Fornito et al., 2013).

Studies identifying graph‐theoretical network differences in BD (Kim et al., 2013; Leow et al., 2013; Roberts et al., 2017; Spielberg et al., 2016) and in MDD (Borchardt et al., 2016; Jin et al., 2011; Lord, Horn, Breakspear, & Walter, 2012; Luo et al., 2015; Meng et al., 2014; Ye et al., 2015; Zhang et al., 2011) have shown promising results distinguishing these patient groups from healthy individuals, implying a disturbed network organization in affective disorders. They predominantly reported alterations of regional topological properties while findings are inconsistent regarding global changes. GT studies comparing both affective disorders are scarce and most of them so far have only focused on currently depressed BD and MDD patients (Lord et al., 2012; Meng et al., 2014; Roberts et al., 2017; Wang, Wang, Jia, Zhong, Zhong et al., 2017; Ye et al., 2015; Zhang et al., 2011). Lately, researchers have investigated state‐dependent differences between not only depressed, but also euthymic individuals suffering from BD and MDD. For example, Sacchet et al. (2015) observed gray matter volume differences in the caudate nucleus for euthymic BD and both depressed and euthymic MDD participants compared to HC, and in the ventral diencephalon between the depressed MDD group and the other three groups. Rive et al. (2016) found different connectivity patterns in the default mode network (DMN) between groups of currently depressed MDD along with remitted MDD and BD individuals.

Studies such as the abovementioned depict the importance of accounting for the patients’ current episode while conducting fMRI studies. To date, BD and MDD patients in an alleviated symptom state are not well examined with r‐fMRI. However, for the interpretation of FC changes in BD and MDD individuals it is necessary to ascertain whether similarities or dissimilarities in FC are caused by the current symptom state or by persistent changes in brain network organization derived from the disorders themselves, regardless of symptom prevalence. This may be fundamental knowledge to understand the pathophysiology of affective disorders. Studies examining emotional behavior have reported disturbed emotion regulation in euthymic BD and remitted MDD and indicated that this might be a risk factor for developing subsequent depressive or manic episodes (Wolkenstein, Zwick, Hautzinger, & Joormann, 2014). Neuroimaging studies focusing on euthymic individuals may therefore help finding neuropathological correlates for these persisting aberrations.

In our current study, we aimed to investigate potential differences in functional brain connectivity in patients with BD and MDD who were in a euthymic state at the time of scan. We used a graph theoretical network analysis approach to analyze the participants’ brain network properties. Network based statistic (NBS), a method based on cluster‐thresholding procedures, was employed to identify subnetworks with altered connectivity patterns between the groups (Zalesky, Fornito, & Bullmore, 2010). We then compared our findings in the two patient groups with a matched group of HC to evaluate whether this approach could distinguish patients with BD and MDD as well as patients and healthy individuals even in a euthymic state of disease.

## 2 METHODS

### 2.1 Participants

We recruited 20 euthymic bipolar patients and 15 euthymic patients with recurrent MDD at the Department of Psychiatry of the Goethe University Frankfurt, Germany. Thirty age‐, gender‐, and education‐matched HC subjects were recruited through local and nationwide newspaper advertisement. All subjects were right‐handed according to the Edinburgh Handedness Inventory (EHI) (Oldfield, 1971) and between 23 and 64 years old. Diagnoses were validated by trained clinicians conducting the Structured Clinical Interview for DSM‐IV disorders Parts I and II (SCID I+II) (First, Gibbon, Spitzer, Williams, & Benjamin, 1997; First, Spitzer, Gibbon, & Williams, 2002). HC were also screened by usage of SCID I and II to ensure that no subject suffered from a psychiatric disease. They had no personal or family history of any psychiatric disorder according to DSM‐IV.

Furthermore, all subjects underwent Beck Depression Inventory II (BDI‐II) (Beck, Steer, & Brown, 1996) and Bech‐Rafaelsen Mania Rating Scale (BRMAS) (Bech, 2002) to assess current depressive and manic symptoms. At the time of participating in the study, all patients were in a euthymic state as determined by BDI‐II values ≤13 and BRMAS values ≤3.

Subjects were excluded if they had a lifetime history of any pathology including psychotic symptoms, substance dependence, neurological illness or if they had any contraindications to magnetic resonance imaging. To reduce the likelihood of MDD patients being misdiagnosed BD patients, we specifically selected individuals with a recurrent course of disease (at least two depressive episodes in the past).

The study was approved by the ethics committee at the medical department of Frankfurt University. All participants gave their written and informed consent prior to take part in the study.

### 2.2 Data acquisition

MR images were collected on a Siemens Magnetom TRIO 3T scanner (Siemens Healthcare, Erlangen, Germany). All subjects underwent a 2D echo planar imaging (EPI) sequence and were instructed to keep their eyes open and fixated on a white dot on black background while thinking of nothing in particular. Scanning lasted 10 min during which we collected 300 volumes, each consisting of 30 axial slices (TR/TE = 2,000/30 ms, slice thickness 3 mm, dist. factor 20%, flip angle 90°, spatial resolution: 3 × 3 × 3 mm, bandwidth: 2,298 Hz/Px).

We used a 3D Modified Driven Equilibrium Fourier Transform (MDEFT) (Deichmann, Schwarzbauer, & Turner, 2004) sequence (176 sagittal slices, TR/TE = 7.91/2.48 ms, TI = 920 ms, slice thickness = 1 mm, dist. Factor 20%, flip angle 16°, spatial resolution 1 × 1 × 1 mm, bandwidth: 195 Hz/Px, scan time: 12 min) to obtain T1 images for reference and to ensure that no subject showed any brain anomalies.

### 2.3 Data processing

Data were preprocessed using DPARSF (Yan, 2010) (RRID:SCR_002372). The first 10 images were discarded to ensure T1 equilibration. Further data processing involved slice timing correction, coregistration of functional and structural data, normalization into Montreal Neurological Institute (MNI) standardized space with a voxel size of 3 × 3 × 3 mm, segmentation of gray matter, white matter and cerebral spine fluid signals and removal of linear trend and bandpass‐filtering (0.01–0.08 Hz) excluding high frequency ranges to capture spontaneous neuronal activity and to remove artifacts induced by physiological processes. We corrected for head motion using the Friston 24 parameter model (Friston, Williams, Howard, Frackowiak, & Turner, 1996; Power et al., 2014). Seven BD, three MDD and six HC subjects from our initial sample of 27 BD, 18 MDD and 36 HC individuals surpassed the predefined head motion threshold of 2 mm translation or 2° rotation in any direction and were therefore excluded. We opted against smoothing our data as this may induce artificial correlations between neighboring voxels (Fornito, Zalesky, & Bullmore, 2010). We also decided not to use global signal regression due to its controversially interpreted effects on FC analysis (Murphy, Birn, Handwerker, Jones, & Bandettini, 2009).

### 2.4 Network construction

For each subject, we defined 90 regions of interest (ROIs) according to the Automatic Anatomic Labelling Atlas (AAL) (Tzourio‐Mazoyer et al., 2002) (RRID:SCR_003550), excluding the cerebellum. We extracted the mean time course for each region and calculated the Pearson coefficients between each pair of ROIs to obtain a 90x90 undirected weighted correlation matrix. Negative weights were converted to zero. Network edges were defined using a sparsity thresholding procedure ranging from 0.1 (i.e. 10% of the strongest connections of the maximum possible number of connections in the network were retained) to 0.5 in steps of 0.01. There is no clear consensus on which network threshold is best suited for examining human brain graphs as a too liberal threshold may result in more frequent false positive connections while a too conservative threshold may elevate the number of false negative connections (Drakesmith et al., 2015). To overcome this issue, we chose to examine each graph metric on these 41 thresholds, ranging from conservative (i.e. 0.1) to liberal (i.e. 0.5).

### 2.5 Network analysis

We chose an array of graph metrics to examine the brain graphs in terms of both global and nodal functional integration and segregation as well as measures of centrality.

#### 2.5.1 Global graph metrics

We applied global clustering coefficient (CC), characteristic path length (PL) and global efficiency (EF) as global graph metrics. Global CC describes the mean value of the fraction of the node's neighbors that are also neighbors to each other and thus illustrates functional segregation, i.e. the capacity of a network for specialized processing in densely interconnected groups of brain regions (Rubinov & Sporns, 2010). We selected characteristic PL and global EF as measures of functional integration. Characteristic PL is the average shortest PL between all pairs of nodes in the network while global EF depicts the average inverse shortest PL.

#### 2.5.2 Nodal graph metrics

Nodal characteristic PL and nodal CC were used to evaluate functional integration and segregation of possibly affected nodes. Low measures of nodal PL resemble higher integration of the concerned nodes in the network, and vice versa. Likewise, more segregated brain network regions are characterized by nodes with higher measures of nodal CC (Rubinov & Sporns, 2010). In addition to nodal PL and CC, we focused on the two most common measures of centrality, degree (DEG) and betweenness centrality (BC). Nodal DEG is defined as the number of links connected to the node. BC represents the fraction of all shortest paths that pass through a respective node. Nodes with high values of BC can be interpreted as hub nodes that integrate divergent parts of the network (Rubinov & Sporns, 2010). All graph metrics were computed using GraphVar (RRID:SCR_014117), a toolbox based on Brain Connectivity Toolbox and Graph Analysis Toolbox (Hosseini, Hoeft, & Kesler, 2012; Kruschwitz, List, Waller, Rubinov, & Walter, 2015; Rubinov & Sporns, 2010).

To evaluate potential associations of illness severity and network aberrations, we calculated Pearson correlation coefficients to analyze possible correlations between symptom rating scales (BDI, BRMAS), illness duration and the graph metrics that exhibited significant between‐group differences.

### 2.6 Statistical analysis

Statistical analyses were conducted with the help of the Statistical Package for the Social Sciences (SPSS, RRID:SCR_002865). Analysis of variance (ANOVA) was performed for between‐group comparison of global and regional network parameters as well as the NBS‐subnetwork analysis explained further below. Prior, we applied linear regression analysis for every ROI to remove potential age and gender influences as covariates. Statistical differences between two groups were further evaluated using post‐hoc two‐sample *t* tests. Additionally, we conducted nonparametric permutation testing (10,000 repetitions) to detect group differences for all global and nodal graph metrics. In each repetition, network measures and ANOVA *F*‐values were randomly reassigned to one of the groups while maintaining the groups' original subject numbers to obtain a permutation distribution. Based on this distribution, *p*‐values were calculated for differences in the actual network measures based on their respective percentile position. We applied false discovery rate (FDR) for multiple comparisons correction (Benjamini & Hochberg, 1995) for all nodal network properties.

Furthermore, we employed NBS to detect subnetworks showing significantly altered connectivity in the patient groups. NBS utilizes nonparametric permutation testing to control the family‐wise error rate (FWER) for topological clusters. This is achieved by arbitrarily choosing a primary test statistic threshold (In our case: *p* < 0.001*/t* = 3.40). Connections exceeding this threshold are summed up to a set of supra‐threshold connections. Among these connections, topological clusters are identified by their respective correlation strengths and compared with the randomly permuted data (10,000 repetitions) to obtain nonparametric *p*‐values for each subnetwork. Further information can be obtained from Zalesky et al. (2010).

Results were visualized with BrainNet viewer (Xia, Wang, & He, 2013) (RRID:SCR_009446).

## 3 RESULTS

### 3.1 Demographic and clinical data

Group comparisons revealed no significant differences in age, gender and mean amount of education years among all three groups. BDI‐II and BRMAS mean scores as well as age of illness onset and illness duration did not differ significantly between BD and MDD individuals. The three participant groups did not differ significantly in BRMAS mean scores. However, significant differences between HC and BD patients as well as between HC and MDD patients were exhibited for BDI‐II mean scores. The majority of the patient sample was taking psychotropic medication (BD: 17/20, MDD: 10/15) at the time of scan. More precisely, 12 BD patients were taking antidepressant pharmaceuticals (MDD: 9), seven were taking neuroleptics (MDD: 2) and 15 were taking mood stabilizing agents (MDD: none). Further demographic and clinical data are depicted in Table 1.

**Table 1:** Demographic and clinical data of all patients with bipolar disorder (BD), major depressive disorder (MDD) and healthy controls (HC)

	BD (*n*=20), (mean ± *SD*)	MDD (*n*=15), (mean ± *SD*)	HC (*n*=30), (mean ± *SD*)	*p* value
Gender (M/F)	10/10	4/11	11/19	0.359^a^
Age	42.60 ± 10.14	41.60 ± 13.69	39.47 ± 13.19	0.667^b^
Education (years)	16.58 ± 1.86	16.07 ± 2.74	16.83 ± 1.95	0.526^b^
Illness onset (years)	27.70 ± 11.16	29.47 ± 14.57	NA	0.687^c^
Illness duration (years)	16.20 ± 11.63	10.00 ± 11.44	NA	0.133^c^
BRMAS	0.50 ± 1.10	0.20 ± 0.78	0.37 ± 0.96	0.665^b^
BDI‐II	5.85 ± 4.83	8.07 ± 4.92	2.60 ± 3.91	<0.001^b^
				0.191^c^ (BD‐MDD)
Medication				
Antidepressant	12 (60%)	9 (60%)		
Neuroleptics	7 (35%)	2 (13%)		
Mood stabilizing	15 (75%)	0		
Sedative	0	0		
No medication	3 (15%)	5 (33%)		

BRMAS: Bech‐Rafaelsen Mania Rating Scale; BDI‐II: Beck Depression Inventory II; NA: not applicable.

a *p*‐values were obtained using a Pearson chi‐squared test.

b *p*‐values were obtained by conducting analyses of variance (ANOVA).

c *p*‐values were obtained using two‐tailed *t* tests.

### 3.2 Differences in global network properties

**Figure 1:**
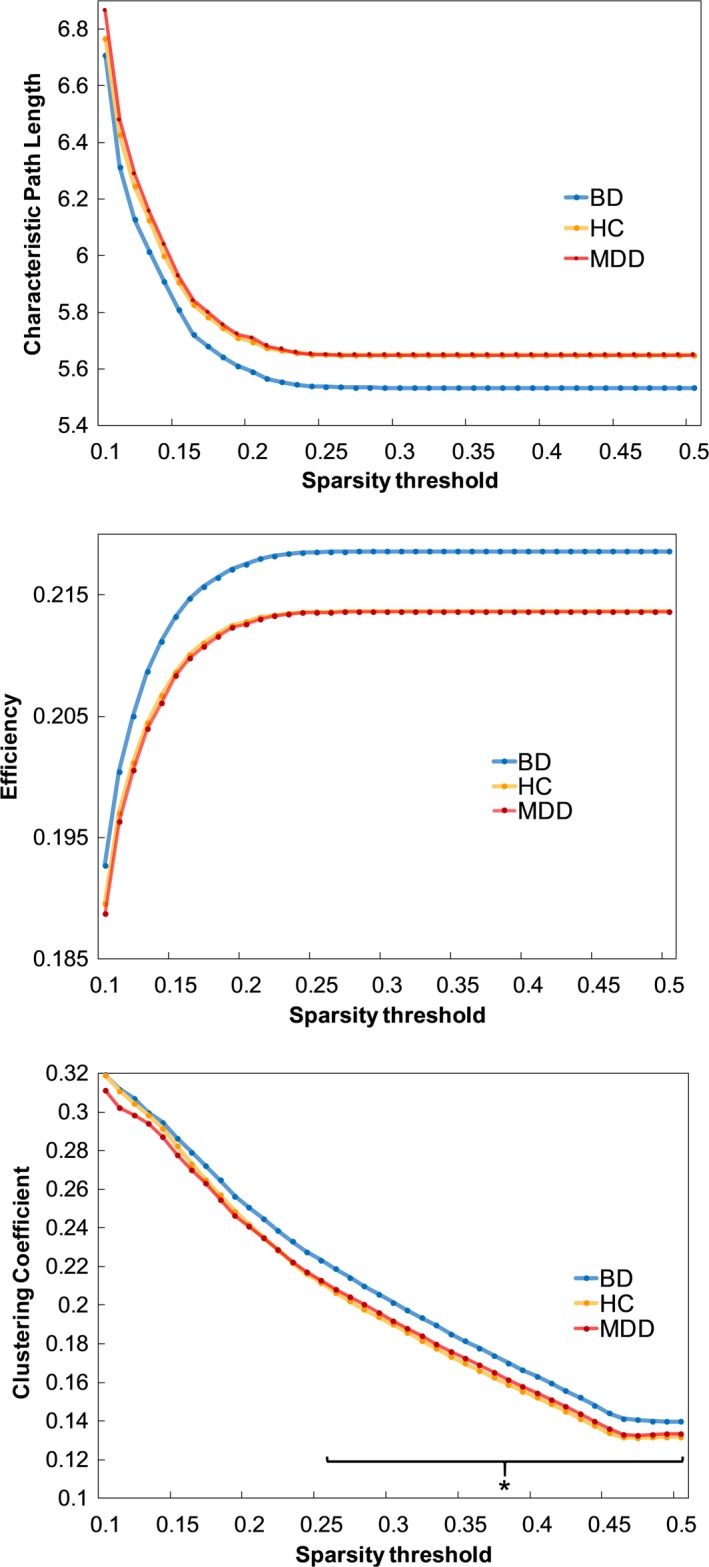
Group differences in global network parameters. The asterisk indicates the observed significantly higher mean global clustering coefficient for BD individuals compared to HC in the sparsity threshold range from 0.26 to 0.50. BD = bipolar disorder; MDD = major depressive disorder; HC = healthy controls

For the global parameters, ANOVA showed a significant group effect in global CC. Post‐hoc analysis revealed that, compared to HC, BD patients exhibited a significantly higher mean global CC in the sparsity threshold range from 0.26 to 0.5 with a maximum at a threshold of 0.47 (*p* = 0.02). These findings did, however, not survive FDR correction. We found no significant group differences in characteristic PL and global EF. No significant differences were found in the MDD group compared to either BD individuals or HC (Figure 1).

### 3.3 Differences in regional network properties

**Figure 2:**
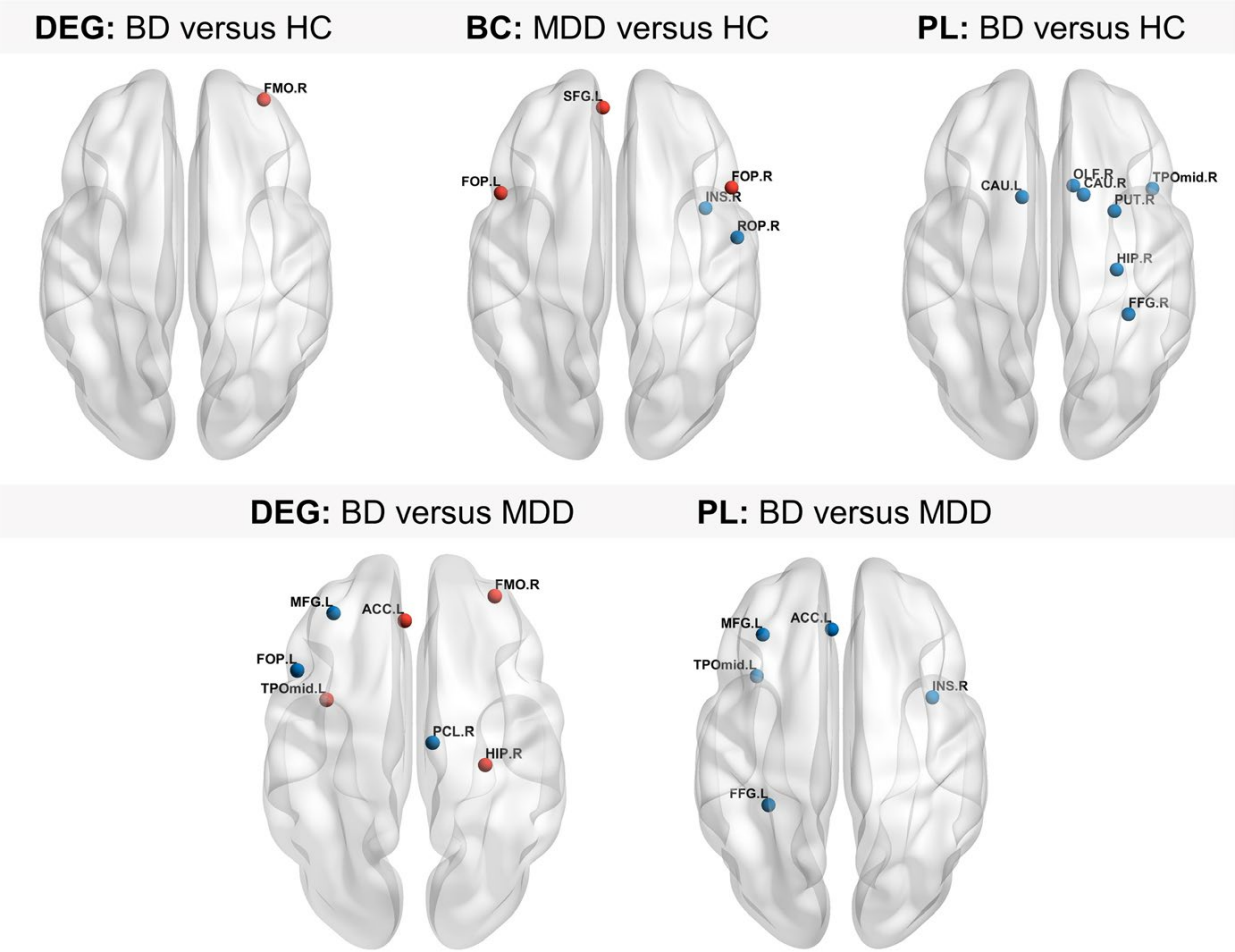
Brain regions showing altered nodal network properties. Between‐group differences in nodal degree, BC and PL as determined by post‐hoc *t* tests Red nodes indicate significantly increased nodal values, blue nodes indicate significantly decreased values (*p* < 0.05, FDR corrected). For abbreviations of the depicted nodes, please consult Table 2. DEG = degree; BC = betweenness centrality; PL = nodal path length

Significant between‐group differences in nodal characteristic PL, DEG and BC are listed in Table 2. Figure 2 illustrates the affected nodes. All nodal differences listed below remained significant after FDR correction. Note that we did not find any between‐group differences in nodal CC that survived the correction.

**Table 2:** Significant between‐group differences in regional network metrics

Betweenness centrality	ANOVA *F*‐value	BD versus HC (*p*)	BD versus MDD (*p*)	MDD versus HC (*p*)
Superior frontal gyrus (SFG) L	2.25	≥0.05	≥0.05	**0.018**
Inf. front. gyrus, opercular (FOP) L	5.89	≥0.05	**0.004**	**0.001**
Inf. front. gyrus, opercular (FOP) R	2.36	≥0.05	≥0.05	**0.025**
Rolandic Operculum (ROP) R	2.94	≥0.05	≥0.05	**0.008**
Insula (INS) R	2.11	≥0.05	≥0.05	**0.027**
Path length				
Middle frontal gyrus (MFG) R	3.81	≥0.05	**0.007**	≥0.05
Olfactory cortex (OLF) R	3.25	**0.002**	≥0.05	≥0.05
Insula (INS) R	4.98	≥0.05	**0.01**	≥0.05
Anterior cingulate cortex (ACC) L	3.66	≥0.05	**0.008**	≥0.05
Hippocampus (HIP) R	2.63	**0.017**	≥0.05	≥0.05
Fusiform gyrus (FFG) L	5.57	≥0.05	**0.004**	≥0.05
Fusiform gyrus (FFG) R	3.12	**0.014**	≥0.05	≥0.05
Caudate nucleus (CAU) L	2.98	**0.003**	≥0.05	≥0.05
Caudate nucleus (CAU) R	3.97	**0.003**	≥0.05	≥0.05
Putamen (PUT) R	4.67	**0.019**	≥0.05	≥0.05
Middle temporal pole (TPO_mid_) L	4.23	≥0.05	**0.001**	≥0.05
Middle temporal pole (TPO_mid_) R	4.63	**<0.001**	≥0.05	≥0.05
Degree				
Middle frontal gyrus (MFG) L	3.35	≥0.05	**0.02**	≥0.05
Inf. front. gyrus, opercular (FOP) L	5.68	≥0.05	**0.007**	≥0.05
Middle frontal gyrus, orbital (FMO) R	9.47	**<0.001**	**<0.001**	≥0.05
Anterior cingulate cortex (ACC) L	4.06	≥0.05	**0.002**	≥0.05
Hippocampus (HIP) R	2.55	≥0.05	**0.01**	≥0.05
Paracentral lobule (PCL) R	2.89	≥0.05	**0.015**	≥0.05
Mid. temp. pole (TPO_mid_) L (T:0.3)	3.95	≥0.05	**0.006**	≥0.05

Notes

All listed regions exhibited significant differences across almost the entire sparsity threshold (*T*) range. Strongest results were most commonly found around a threshold of 0.35. All values displayed were measured on *T* = 0.35, except for DEG values of the left TPO_mid_ which only remained significant in a threshold range from 0.12 to 0.30. Bold font indicates significant differences in post‐hoc *t* tests (*p* < 0.05, FDR corrected).

ANOVA: analysis of variance; BD: bipolar disorder; MDD: major depressive disorder; HC: healthy control.

Significant ANOVA‐group effects were found for nodal PL in the right hippocampus (HIP), right putamen (PUT), left and right caudate nuclei (CAU), left and right fusiform gyri (FFG), right olfactory cortex (OLF), left and right middle temporal poles (TPO_mid_), right middle frontal gyrus (MFG), right insula (INS) and the left anterior cingulate cortex (ACC). We found significant group effects for DEG in the left MFG, left opercular part of the inferior frontal gyrus (FOP), right orbital part of the middle frontal gyrus (FMO), left ACC, right HIP, right paracentral lobule (PCL) as well as the left TPO_mid_, and for BC in the left superior frontal gyrus (SFG), left and right FOP, right Rolandic operculum (ROP) and right INS.

Post‐hoc analysis of DEG revealed significantly higher values in the right FMO in BD patients compared to HC. The BD group further showed significantly lower nodal PL in the right HIP, right PUT, left and right CAU, right FFG, right OLF and the right TPO_mid_.

Compared to MDD individuals, the BD group showed significantly lower BC in the left FOP. Significantly lower values of DEG were found in left FOP, left MFG and right PCL while higher values were found in the right FMO, left ACC, left TPO_mid_ and right HIP. Furthermore, BD patients displayed significantly decreased nodal PL in the right MFG, right INS, left ACC, left FFG and in the left TPO_mid_.

Differences between MDD patients and HC after FDR correction were solely found in BC. Specifically, we found significantly higher BC values in the left and right FOP and the left SFG and significantly lower measures of BC in the right INS and the right ROP.

We found no significant correlations that survived FDR correction between significant graph metrics, symptom rating scales and illness duration in the patient groups.

### 3.4 Network based statistics

**Figure 3:**
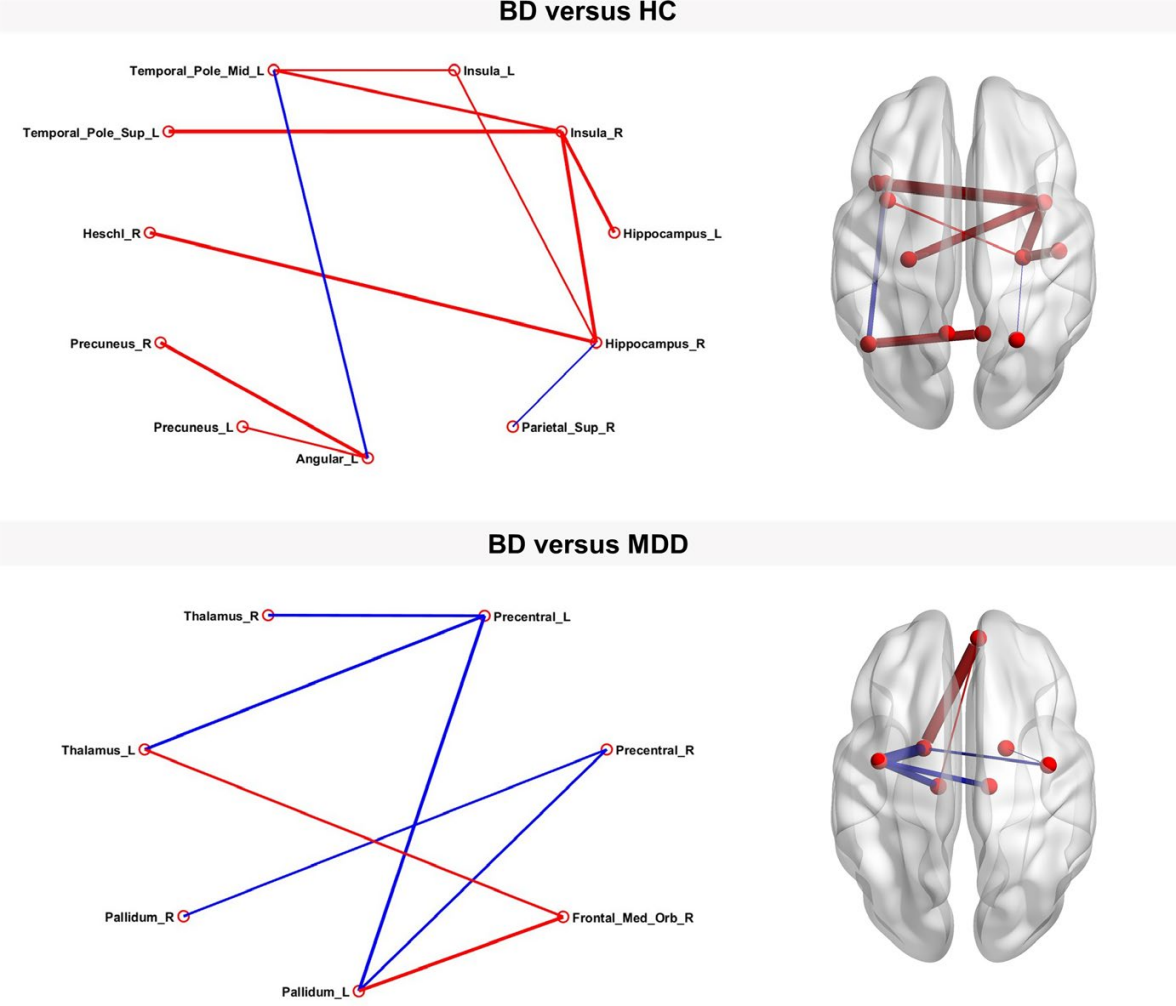
Subnetworks detected by network based statistic analysis. Networks with significantly altered connectivity between bipolar disorder individuals compared to healthy controls (*p* < 0.001, family‐wise error corrected) and major depressive disorder individuals (*p* = 0.003, family‐wise error corrected)**. **Red lines indicate significantly increased connectivity strength between the connected nodes, blue lines indicate significantly decreased connectivity. Density of lines indicates effect size

We found a subnetwork consisting of 11 nodes that showed significantly increased connection strengths mostly in bilateral temporal regions in the BD group compared to HC. Comparing BD with MDD individuals, we found a significantly altered network comprising seven nodes with predominantly increased connectivity in fronto‐subcortical connections but decreased connectivity in parieto‐subcortical links involving the bilateral thalamus as well as the bilateral globus pallidus. Further information about the nodes constituting the subnetworks may be obtained from Figure 3.

## 4 DISCUSSION

We detected abnormalities in both global and regional network organization distinguishing euthymic BD patients, euthymic patients with recurrent MDD and HC. At the global level, the BD patients showed a significantly higher mean global CC compared to the HC which did not remain significant after FDR correction. In our analysis of regional network properties, we detected mainly temporal and subcortical nodes exhibiting significant discrepancies in network integration in the BD patient group compared to HC. Likewise, we primarily identified nodes with altered network integration and centrality when comparing BD to MDD patients. The MDD and HC groups only differed in BC in frontal and temporal nodes. Global differences and differences in other nodal parameters were mostly prevalent when comparing the BD group with either HC or MDD participants. This tendency also applied for our NBS analysis in which we found a significantly altered predominantly temporal subnetwork when comparing BD patients to HC. NBS analysis of BD versus MDD individuals revealed a subnetwork with altered fronto‐subcortical and parieto‐subcortical connections, while no significant differences in brain connectivity were found between MDD patients and HC. To our knowledge, this is the first study to investigate graph analytical functional network properties in euthymic BD and euthymic MDD patients. Our results may therefore shed light on the underlying neuropathological correlates of both affective disorders in the observed alleviated state of clinical symptoms.

We found a significantly higher mean global CC value in BD compared to the HC on the global network level. This implicates an increased amount of functional interconnectivity in the average BD brain network. This result did not remain significant after FDR correction and we were not able to find any differences in nodal clustering coefficient between the groups. Hence, the observed effect appears to be spread over the whole brain network and could not be tracked down to a specific set of nodes. Overall, comparing our results to other studies utilizing graph theory to examine differences between BD and MDD is difficult as these studies are still rare and results among them vary. While a previous study reported a significantly higher global CC mean value (He et al., 2016) between a group of BD patients in mixed states (mainly depressed) and a group of acutely depressed MDD patients, another study comparing depressed BD and MDD individuals (Wang, Wang, Jia, Zhong, Zhong et al., 2017) did not detect any significant differences in global CC. They instead reported significantly higher global PL and lower EF mean values for both acutely depressed BD and MDD patients compared to HC. The only other study analyzing graph theoretical measures in a sample of euthymic BD patients used structural brain network data derived from DTI sequences and found a lower mean CC, lower EF and higher characteristic PL when comparing their data to HC (Leow et al., 2013). Diverging results of global network parameters may also be found in studies investigating MDD patients. For example, Zhang et al. (2011) found higher mean global EF and lower characteristic PL for first‐episode MDD patients versus HC whereas others did not find any differences on the global network level (Lord et al., 2012; Peng et al., 2014; Ye et al., 2015). There are several possible reasons for these inconsistencies: differing patient samples (age, gender, medication, illness duration, illness severity), method (structural versus functional connectivity), choice of brain atlas (Cao et al., 2014) and network generation (Andellini, Cannatà, Gazzellini, Bernardi, & Napolitano, 2015) to name only a few possible confounds. Clearly, there is more need for further studies to address these issues.

In the regional network connectivity analysis, we found differences between the BD group and HC mostly in nodal centrality parameters in frontal regions along with alterations of nodal PL in temporal and subcortical regions associated to the limbic system. Bilateral temporal and subcortical regions likewise showed abnormalities in the NBS analysis. We observed elevated measures of DEG in the right FMO in BD compared to HC, a node located in the orbitofrontal cortex (OFC). The OFC is associated with functions such as emotion regulation. Thus, aberrations in the OFC cause dysregulated emotional responses which may possibly lead to pathological mood states in BD (Savitz, Price, & Drevets, 2014). Favre, Baciu, Pichat, Bougerol, and Polosan (2014) conducted a seed‐based correlation analysis in which they reported increased FC between the prefrontal cortex (PFC) and the limbic system in a comparison between euthymic BD individuals and HC. They concluded that the increased connectivity may reflect an excessive attentional focus on emotions persisting in a euthymic state. This promotes the assumption of residual symptoms such as mood lability and increased emotional reactivity in euthymic individuals with BD, which can be further reinforced by our results depicting a prefrontally located node exhibiting a significantly elevated DEG.

Comparing BD to HC, we detected significant reductions of nodal PL in the BD sample exclusively in temporal regions (right FFG and TPO_mid_), right HIP and right and left CAU, all associated with the limbic circuitry as a central component of emotion processing. These findings indicate that the aforementioned nodes are more integrated into the brain network of BD patients than in networks of individuals not suffering from BD, possibly leading to a disturbed perception of emotions in BD. Changes in the limbic system supporting our results were both found in resting‐state paradigms (Ambrosi et al., 2017; Anand et al., 2009; Lois, Linke, & Wessa, 2014; Wang, Wang, Jia, Zhong, Zhong et al., 2017) as well as in task‐based paradigms (Gruber et al., 2010; Strakowski, Adler, Holland, Mills, & DelBello, 2004; Thermenos et al., 2010; Townsend & Altshuler, 2012). In most studies, irregularities were registered in the frontal lobes along with temporal and subcortical regions such as hippocampus and basal ganglia. In contrast to our findings, many studies reported aberrations in the amygdala as a key component of the limbic network. Hyperactivation in the amygdala was not only reported in task‐based fMRI studies presenting emotionally arousing pictures (Townsend & Altshuler, 2012) but also in prior examinations of resting‐state FC: Previous research indicated compromised FC especially between the amygdala and prefrontal regions in acutely depressed BD patients (Li et al., 2015; Liu et al., 2015; Spielberg et al., 2016). However, most studies examining euthymic BD individuals did not report any significant effects in amygdala FC, consistent with our results (Townsend & Altshuler, 2012). Instead, they depicted changes in euthymic patients compared to healthy individuals most commonly in frontal areas, limbic regions such as the temporal cortex as well as the hippocampus and, concordant to our findings, in striatal regions including the caudate nucleus. Our findings thus support the presumption that abnormalities in the amygdala appear to be more prevalent in acute mood states while deviances in the frontal lobes and the limbic system (excluding the amygdala) such as our findings in the temporal cortex and the hippocampus are present in BD patients regardless of illness state.

In the comparison between the BD and the MDD group, affected nodes were mostly found frontal, temporal and in subcortical regions such as the hippocampus and the basal ganglia. Within the frontal regions, some nodes exhibited higher measures of centrality (left ACC, right FMO) while others (left MFG, left FOP) displayed significantly lower BC and DEG for BD compared to MDD. BD patients additionally exhibited higher values of DEG in the left TPO_mid_ and the right hippocampus compared to the MDD. We located nodes with significantly lower nodal PL frontal (right MFG, left ACC) and temporal (right INS, left FFG, left TPO_mid_). These nodes with lower PL were often accompanied by significantly higher values of BC and/or DEG. Hence, affected regions in BD patients seem to be more central and integrated into the whole brain graph compared to MDD. Likewise, this finding applied to the ACC which also presented low nodal PL along with high BC values. The affected frontal regions including the ACC are involved in introspection and rumination and are not only reported to be affected in BD but also in MDD patients (Cooney, Joormann, Eugène, Dennis, & Gotlib, 2010). He et al. (2016) identified regions in the PFC and ACC that differed between acute BD and MDD patients. Specifically, the BD group showed significantly stronger FC strengths within the prefrontal cortex as well as between prefrontal cortex and ACC, cuneus and the superior temporal and parahippocampal gyrus. Our results imply that network centrality and integration in the aforementioned regions remain elevated in a euthymic state of BD compared to euthymic MDD. Patients with BD may therefore be more afflicted by ruminative thoughts than MDD patients in the absence of a depressive episode.

In an fMRI study utilizing an emotion regulation paradigm, Rive et al. (2015) examined depressed and remitted BD and MDD patients and found a significant impairment in emotion regulation in the examined BD sample. While there were no significant differences in emotion regulation between remitted MDD and HC, remitted BD showed impaired emotion regulation corresponding with an increased activity in frontal regions in remitted BD compared to remitted MDD (Rive et al., 2015). Our results regarding higher measures of centrality in conjunction with lower nodal PL in frontal areas may be the GT‐correlate of previous findings distinguishing symptomless BD and MDD as conducted by Rive et al. (2015). We thus reinforce their proposition that impairments of emotion processing persist in BD but less in MDD during remission.

Functional connectivity alterations in the temporal lobes between BD and MDD have been consistently reported by prior studies (He et al., 2016; Rive et al., 2016; Wang, Wang, Jia, Zhong, Niu et al., 2017; Wang, Wang, Jia, Zhong, Zhong et al., 2017). Interestingly, previous structural MRI studies have reported decreased cortical thickness in temporal as well as frontal areas including the ACC in individuals with BD (Hanford, Nazarov, Hall, & Sassi, 2016). Wang, Wang, Jia, Zhong, Niu et al. (2017) examined a patient sample of currently depressed BD and MDD and proposed that their findings of an increased long‐range functional connectivity strength in the middle temporal gyrus in BD may display a compensatory mechanism to account for the impairments in gray matter structure (Wang, Wang, Jia, Zhong, Niu et al., 2017). We second this proposition and further hypothesize that the pattern we found in the frontal and temporal regions of our BD sample (high measures of centrality alongside low PL) may indicate structural deficits in these areas which the brain tries to compensate through a denser, more integrated functional organization.

By conducting NBS analysis, we revealed aberrant connectivity in a network comprising the bilateral thalamus, pallidal nodes as well as prefrontal and parietal cortical nodes in BD compared to MDD. This affected network matches with the limbic‐cortical‐striatal‐pallidal‐thalamic loop, a neural circuit known to partake in emotional behavior, cognitive performance alongside other regulation and response mechanisms associated with mood disorders (Drevets, Price, & Furey, 2008; Sheline, 2000). In a previous seed‐based FC study with acutely depressed MDD as well as depressed and manic BD participants, decreases of corticolimbic connectivity were found in both BD and MDD patients compared to HC with more distinct differences in the BD group (Anand et al., 2009). The orbito‐frontally located FMO was not only part of this subnetwork but also exhibited significantly higher values of DEG in the comparison between both BD and MDD as well as BD and HC. Aberrancies in the OFC potentially lead to impulsivity and euphoria which are characteristic symptoms of manic episodes (Savitz et al., 2014). Since the FMO showed robust differences between BD and the other two groups, it may be a promising marker for detecting BD or distinguishing BD patients from those with MDD.

Comparing the MDD patients to HC, we identified significantly higher BC values in frontal areas (left SFG, right and left FOP) alongside lower BC in the right INS and ROP. The insula with its connections to the fronto‐limbic network plays a key role in emotionally interpreting sensory information. Aberrations in the insular cortex may lead to misinterpretation of emotional stimuli (Sliz & Hayley, 2012). Lower insular BC may represent a disconnection from the brain network leading to disturbed emotional information processing. Similar evidence was reported in preceding studies investigating FC in MDD. For example, Guo et al. (2015) conducted a seed‐based analysis of the insula in drug‐naïve, acutely depressed MDD patients. They reported significantly decreased FC between the insula and frontal, temporal and occipital gyri. Previous studies applying graph analytic measures to investigate MDD patients consistently presented distinctions of BC in frontal and temporal regions compared to HC (Lord et al., 2012; Luo et al., 2015; Meng et al., 2014; Ye et al., 2015; Zhang et al., 2011), making BC an interesting nodal parameter for further evaluation in succeeding GT studies with MDD patients.

Besides frontal and insular aberrations in BC values, we found no further areas with significantly altered nodal parameters differentiating MDD and HC. This could implicate that BD compared to MDD involves more extensive residual changes in network organization in a euthymic state while most nodal parameters in MDD are closer to a healthy state in euthymia. Another possible hypothesis is based on previous studies that found comparable FC changes in depressed BD and MDD patients (for reference, see e.g. Anand et al., 2009 and Wang, Wang, Jia, Zhong, Zhong et al., 2017): Although both disorders have similar effects on FC in the limbic system, BD subjects show more severe changes in brain network organization. Effects in MDD are less severe and could therefore not be registered in a euthymic state.

To date, there is a lack of related studies containing both euthymic BD and MDD samples. Most results discussed here therefore had to be compared to GT studies who either only examined one type of affective disorder or with study samples of acutely depressed BD and MDD. In a GT study using a methodology similar to ours, acutely depressed BD and MDD shared many similarities in global and nodal FC aberrations compared to HC (Wang, Wang, Jia, Zhong, Zhong et al., 2017). At the global network level, both depressed BD and MDD exhibited increased PL and reduced EF compared to HC. Similarities in nodal network parameters were found in the right and left superior frontal gyri and the left middle cingulum where both BD and MDD exhibited a significantly lower nodal EF than the HC group. In their modularity analysis, Wang, Wang, Jia, Zhong, Zhong et al. (2017) found the global values in the limbic network for CC and EF to be significantly decreased in both MDD and BD with a significant increase of PL in both affective disorder samples compared to HC. In contrast to the results of Wang, Wang, Jia, Zhong, Zhong et al. (2017) we found no shared brain network abnormalities between our euthymic BD and MDD samples. Hence, network differences between these disorders might be overshadowed by the clinical condition the patients are experiencing which, in case of a depressive episode, could present a comparable pattern of resting‐state FC aberrations regardless of the underlying disorder. If it can be confirmed that BD involves more residual alterations in network organization, examining these patient groups in a euthymic state will possibly facilitate their distinction. This can, however, not be affirmed by our study due to its cross‐sectional design and needs to be further investigated by subsequent inquiries.

Some limitations of our study need to be further displayed. First, the sample size was relatively small, especially with regard to the MDD group. There is a possibility that our discrepant findings in both affective disorder groups (less differences between MDD and HC compared to BD versus HC) may have been caused by the lower sample size in the MDD group. To address this issue, we conducted a subanalysis in which we excluded five BD patients at random to attain equal sample sizes in both groups. The findings in global and nodal parameters remained similar. Most patients were taking medication at the time of scanning. Hence, there is a possibility that the between‐group analysis was influenced by the usage of different substances such as mood stabilizers in BD and MDD. However, choosing to study only unmedicated patients may lead to a possible bias toward individuals with less severe courses of illness, therefore making it an unrealistic representation of the chronically affected BD and recurrent MDD population (Phillips, Travis, Fagiolini, & Kupfer, 2008). Furthermore, medication effects are believed to have a normalizing effect (i.e. diminishing differences between BD and HC) on FC aberrations in BD patients (Hafeman, Chang, Garrett, Sanders, & Phillips, 2012) which makes it unlikely that our effects were caused by medication usage. Additional knowledge on the effects of medication in graph theoretical analysis of patients with mood disorders is needed to better evaluate whether certain GT parameters are modulated by different classes of neuro‐pharmaceuticals. It has been shown that the choice of brain atlas might influence graph analytical results (Cao et al., 2014). Since we used only one, relatively coarse brain template we cannot draw any definitive conclusions based on our data without subsequent studies confirming our results. This also means that our results should be compared with caution to other studies using a different brain atlas. We also did not acquire data from acutely depressed individuals to compare with our results. We therefore suggest that future studies should include both remitted and acutely affected subjects to evaluate which of the reported effects are truly state‐independent. A common problem arising from a cross‐sectional study design is the possibility of individuals diagnosed with MDD later converting to BD (Dudek, Siwek, Zielińska, Jaeschke, & Rybakowski, 2013). This issue could be avoided or minimized by resorting to a longitudinal study design which may also be applied to examine the same subjects in different mood states.

## 5 CONCLUSION

In this study, we were able to successfully detect graph theoretical parameters separating patients with BD from MDD patients and HC participants. The presented results indicate aberrations of resting‐state network topology in euthymic BD in the frontal and temporal cortex. Concerned regions were mostly part of the limbic circuitry. We demonstrated that BD and MDD patients in a euthymic state exhibit differences in brain network properties in these regions. These findings may illustrate the neuropathological correlates of persisting changes in emotional information processing distinguishing euthymic BD from euthymic MDD patients. We therefore suggest that graph analyses of FC data could be further implemented by subsequent research projects to evaluate the utilization of this procedure as a possible biomarker eligible to not only separate BD from healthy but also from unipolar depressed individuals.

## DISCLOSURES

MRI data were acquired at Brain Imaging Center Frankfurt, supported by the German Research Council (DFG) and the German Ministry for Education and Research (BMBF; Brain Imaging Center Frankfurt/Main, DLR 01GO0203).

## CONFLICT OF INTEREST

The authors declare no biomedical financial interests or potential conflicts of interest.
