# Supplementary material for: Aberrant brain network topology in fronto‐limbic circuitry differentiates euthymic bipolar disorder from recurrent major depressive disorder
Source: Brain Behav. 2019 May 7;9(6):e01257. doi: 10.1002/brb3.1257 (PMC6576154; doi:10.1002/brb3.1257)
Supplement: Supplementary file 1 [file BRB3-9-e01257-s001.docx]

**Supplementary Material for:**

**Aberrant brain network topology in fronto-limbic circuitry differentiates euthymic bipolar disorder from recurrent major depressive disorder**

Jannis Dvorak, Marietheres Hilke, Marco Trettin, Sofia Wenzler, Marleen Hagen, Naddy Ghirmai, Maximilian Müller, Dominik Kraft, Andreas Reif, Viola Oertel

**Network analysis**

Several regional and global network metrics were derived from weighted, undirected connectivity matrices and are based on a graph *G = (N, K)* where *N* is the set of all nodes in the network and *K* is the set of all network edges.

*Regional network metrics*

Four regional network metrics were examined: degree (*k*), betweenness centrality (*b*), nodal characteristic path length (*L_i_*) and nodal clustering coefficient (*C_i_*).

The degree of a node is defined as: $k_{i}=\sum_{j\in N} w_{ij}$ , where *w_ij_* is the weighted connection status between nodes *i* and *j*.

Betweenness centrality is measured as:

$$b_{i}=\begin{aligned} \sum_{h,j\in N} \\ h\neq j,h\neq i,j\neq i, \end{aligned}\frac{\rho_{hj}(i)}{\rho_{hj}}$$

where *ρ_hj_* resembles the number of shortest paths between nodes *h* and *j* while *ρ_hj_(i)* is the number of shortest paths between *h* and *j* passing through *i*.

The nodal characteristic path length (i.e. mean shortest path length of a node) depicts the mean shortest path length between a node *i* and all other nodes in the network:

$$L_{i}=\frac{1}{n-1}\sum_{i\neq j\in G} d_{i,j}$$

where *d_ij_* is the shortest path length from node *i* to node *j*.

Nodal clustering coefficient is defined as:

$$C_{i}=\frac{1}{n}\sum_{i\in N} \frac{2t_{i}}{k_{i}(k_{i}-1)}$$

where *t_i_* represents the number of triangular connections around a node *i* and *k_i_* is the degree of a node *i*.

$$t_{i}=\frac{1}{2}\sum_{j\text{,}h\in N} w_{ij}w_{ih}w_{jh}$$

*Global network metrics*

We examined global characteristic path length (*L*), global efficiency (*E*) and global clustering coefficient (*C*) as global parameters. Global characteristic path length is defined as:

$$L^{\text{ }}=\frac{1}{n}\sum_{i\in N} \frac{\sum_{j\in N\text{,}j\neq i} d_{ij}}{n-1}$$

Global efficiency depicts the average inverse characteristic path length and therefore is defined as:

$$E^{\text{ }}=\frac{1}{n}\sum_{i\in N} \frac{\sum_{j\in N\text{,}j\neq i} {(d_{ij})}^{-1}}{n-1}$$

Global clustering coefficient represents the sum of all nodal clustering coefficients:

$$C=\frac{1}{n}\sum_{i\in N} C_{i}=\frac{1}{n}\sum_{i\in N} \frac{2t_{i}}{k_{i}(k_{i}-1)}$$

**Sub-analysis results**

As mentioned in the limitations section, we conducted a sub-analysis to attain equal sample sizes in both disorder groups. We excluded five BD subjects at random and analysed the global and nodal GT parameters of this sub-group consisting of 15 BD, 15 MDD and 30 HC individuals.

For global network parameters, we only found significant differences in global CC at a threshold range from 0.44 to 0.46. Post-hoc analysis revealed a barely-significant increase in global CC in the BD group compared to HC with a maximum at a threshold of 0.45 (*p*= 0.46) with a trend towards an increased global CC (*p* between 0.05 and 0.1) at the threshold range from 0.33 to 0.48. No significant group effects were found for characteristic PL and global EF.

Results of the sub-analysis for the nodal network parameters may be found in Table S2.

**References**

Achard, S., & Bullmore, E. (2007). Efficiency and cost of economical brain functional networks. *PLoS Computational Biology*, *3*(2), e17. https://doi.org/10.1371/journal.pcbi.0030017

Rubinov, M., & Sporns, O. (2010). Complex network measures of brain connectivity: Uses and interpretations. *NeuroImage*, *52*(3), 1059–1069. https://doi.org/10.1016/j.neuroimage.2009.10.003

Tzourio-Mazoyer, N., Landeau, B., Papathanassiou, D., Crivello, F., Etard, O., Delcroix, N., … Joliot, M. (2002). Automated anatomical labeling of activations in SPM using a macroscopic anatomical parcellation of the MNI MRI single-subject brain. *NeuroImage*, *15*(1), 273–289. https://doi.org/10.1006/nimg.2001.0978

Watts, D. J., & Strogatz, S. H. (1998). Collective dynamics of “small-world” networks. *Nature*, *393*(6684), 440–2. https://doi.org/10.1038/30918

**Table S1.** Brain regions as determined by the Automated Anatomical Labeling (AAL, Tzourio-Mazoyer et al., 2002) parcellation procedure and their respective indices and abbreviations.

| **Index** | **Brain Region** | **Abbr.** | **Index** | **Brain Region** | **Abbr.** |
| --- | --- | --- | --- | --- | --- |
| 1,2 | Precentral gyrus | PreCG | 47, 48 | Lingual gyrus | LING |
| 3,4 | Superior frontal gyrus, dorsolateral | SFG | 49, 50 | Superior occipital lobe | SOL |
| 5,6 | Superior frontal gyrus, orbital | SFGorb | 51, 52 | Middle occipital lobe | MOL |
| 7,8 | Middle frontal gyrus | MFG | 53, 54 | Inferior occipital lobe | IOL |
| 9,10 | Middle frontal gyrus, orbital | FMO | 55, 56 | Fusiform gyrus | FFG |
| 11,12 | Inferior frontal gyrus, opercular | FOP | 57, 58 | Postcentral gyrus | PostCG |
| 13,14 | Inferior frontal gyrus, triangular | FTR | 59, 60 | Superior parietal lobe | SPL |
| 15, 16 | Inferior frontal gyrus, orbital | FOR | 61, 62 | Inferior parietal lobe | IPL |
| 17, 18 | Rolandic operculum | ROP | 63, 64 | Supramarginal gyrus | SMG |
| 19, 20 | Supplementary motor area | SMA | 65, 66 | Angular gyrus | ANG |
| 21, 22 | Olfactory cortex | OLF | 67, 68 | Precuneus | PreCUN |
| 23, 24 | Superior frontal gyrus, medial | SFM | 69, 70 | Paracentral lobule | PCL |
| 25, 26 | Superior frontal gyrus, medial orbital | SFMorb | 71, 72 | Caudate nucleus | CAU |
| 27, 28 | Gyrus rectus | REC | 73, 74 | Putamen | PUT |
| 29, 30 | Insula | INS | 75, 76 | Globus pallidus | PAL |
| 31, 32 | Cingulate gyrus, anterior part | ACC | 77, 78 | Thalamus | THA |
| 33, 34 | Cingulate gyrus, mid part | MCC | 79, 80 | Transverse temporal gyrus (Heschl) | HES |
| 35, 36 | Cingular gyrus, posterior part | PCC | 81, 82 | Superior temporal gyrus | STG |
| 37, 38 | Hippocampus | HIP | 83, 84 | Superior temporal pole | TPOsup |
| 39, 40 | Parahippocampal gyrus | PHG | 85, 86 | Middle temporal gyrus | MTG |
| 41, 42 | Amygdala | AMY | 87, 88 | Middle temporal pole | TPOmid |
| 43, 44 | Calcarine sulcus | CAL | 89, 90 | Inferior temporal gyrus | ITG |
| 45, 46 | Cuneus | CUN |  |  |  |

**Table S2.** Between-group differences in regional network metrics in sub-analysis with equal sample sizes.

| **Betweenness Centrality** | **ANOVA *F*-value** | **BD vs HC (*p*)** | **BD vs MDD (*p*)** | **MDD vs HC (*p*)** |
| --- | --- | --- | --- | --- |
| Superior frontal gyrus (SFG) L | 2.58 | ≥ 0.05 | **0.019** | **0.016*** |
| Inf. front. gyrus, opercular (FOP) L | 8.19 | ≥ 0.05 | **0.002*** | **0.001*** |
| Inf. front. gyrus, opercular (FOP) R | 1.73 | ≥ 0.05 | ≥ 0.05 | **0.026** |
| Rolandic Operculum (ROP) R | 2.89 | ≥ 0.05 | ≥ 0.05 | **0.01*** |
| Insula (INS) R | 2.32 | ≥ 0.05 | **0.009*** | **0.024** |
|  |  |  |  |  |
| **Path Length** |  |  |  |  |
| Middle frontal gyrus (MFG) R | 3.13 | ≥ 0.05 | ≥ 0.05 | ≥ 0.05 |
| Olfactory cortex (OLF) R | 3.17 | **0.002*** | ≥ 0.05 | ≥ 0.05 |
| Insula (INS) R | n.s. | ≥ 0.05 | ≥ 0.05 | ≥ 0.05 |
| Anterior cingulate cortex (ACC) L | 4.27 | ≥ 0.05 | **0.007*** | ≥ 0.05 |
| Hippocampus (HIP) R | n.s. | ≥ 0.05 | ≥ 0.05 | ≥ 0.05 |
| Fusiform gyrus (FFG) L | 5.78 | **0.002*** | **0.002*** | ≥ 0.05 |
| Fusiform gyrus (FFG) R | 4.53 | **0.002*** | ≥ 0.05 | ≥ 0.05 |
| Caudate nucleus (CAU) L | 2.98 | **0.001*** | ≥ 0.05 | ≥ 0.05 |
| Caudate nucleus (CAU) R | 3.86 | **0.005*** | ≥ 0.05 | ≥ 0.05 |
| Putamen (PUT) R | n.s. | ≥ 0.05 | ≥ 0.05 | ≥ 0.05 |
| Middle temporal pole (TPOmid) L | 2.71 | ≥ 0.05 | **0.01*** | ≥ 0.05 |
| Middle temporal pole (TPOmid) R | 3.19 | **0.007*** | ≥ 0.05 | ≥ 0.05 |
|  |  |  |  |  |
| **Degree** |  |  |  |  |
| Middle frontal gyrus (MFG) L | n.s. | ≥ 0.05 | ≥ 0.05 | ≥ 0.05 |
| Inf. front. gyrus, opercular (FOP) L | 5.56 | ≥ 0.05 | **0.008*** | ≥ 0.05 |
| Middle frontal gyrus, orbital (FMO) R | 10.36 | **<0.001*** | **<0.001*** | ≥ 0.05 |
| Anterior cingulate cortex (ACC) L | 5.16 | ≥ 0.05 | **0.001*** | ≥ 0.05 |
| Hippocampus (HIP) R | n.s. | ≥ 0.05 | ≥ 0.05 | ≥ 0.05 |
| Paracentral lobule (PCL) R | n.s. | ≥ 0.05 | ≥ 0.05 | ≥ 0.05 |
| Mid. temp. pole (TPOmid) L (T:0.3) | 3.28 | ≥ 0.05 | **0.032** | ≥ 0.05 |

Results of our sub-analysis with 15 BD, 15 MDD patients and 30 HC. Five BD patients were randomly excluded from our original sample to attain equal sample sizes in both affective disorder groups. All listed regions exhibited significant differences across almost the entire sparsity threshold (T) range. As in the main analysis, all values displayed were measured on T=0.35, except for DEG values of the left TPOmid which only remained significant in a threshold range from 0.12 to 0.30. Bold font indicates significant differences in post-hoc *t*-tests (*p*<0.05). Differences marked with an asterisk (*) survived FDR-correction and display the already corrected p-values.

ANOVA: analysis of variance, BD: bipolar disorder, MDD: major depressive disorder, HC: healthy control, n.s.: not significant
